# Using Live-Cell Imaging and Synthetic Biology to Probe Directed Migration in *Dictyostelium*

**DOI:** 10.3389/fcell.2021.740205

**Published:** 2021-10-05

**Authors:** Jonathan Kuhn, Yiyan Lin, Peter N. Devreotes

**Affiliations:** Department of Cell Biology, School of Medicine, Johns Hopkins University, Baltimore, MA, United States

**Keywords:** migration, chemotaxis, signaling, imaging, synthetic & systems biology

## Abstract

For decades, the social amoeba *Dictyostelium discoideum* has been an invaluable tool for dissecting the biology of eukaryotic cells. Its short growth cycle and genetic tractability make it ideal for a variety of biochemical, cell biological, and biophysical assays. Dictyostelium have been widely used as a model of eukaryotic cell motility because the signaling and mechanical networks which they use to steer and produce forward motion are highly conserved. Because these migration networks consist of hundreds of interconnected proteins, perturbing individual molecules can have subtle effects or alter cell morphology and signaling in major unpredictable ways. Therefore, to fully understand this network, we must be able to quantitatively assess the consequences of abrupt modifications. This ability will allow us better control cell migration, which is critical for development and disease, *in vivo*. Here, we review recent advances in imaging, synthetic biology, and computational analysis which enable researchers to tune the activity of individual molecules in single living cells and precisely measure the effects on cellular motility and signaling. We also provide practical advice and resources to assist in applying these approaches in *Dictyostelium*.

## Introduction

### *Dictyostelium* as a Model Organism for Directed Migration

In order to respond to cues in their environment, cells detect and move toward or away from cues like light, chemical gradients, and mechanical forces. Directed migration is a critical process in all domains of life: unicellular organisms can detect and move toward food and mates, while multicellular organisms use chemical gradients to guide cells during development and immune response (Yamada and Cukierman, 2007; Thomas et al., 2018; Yamada and Sixt, 2019). Understanding how cells sense direction and respond is therefore important both to basic biological questions and for developing new therapies to treat birth defects, immunodeficiencies, and cancers.

Directed migration consists of three processes: directional sensing, motility and polarity. Directional sensing is the orientation of molecules involved in cell migration toward a stimulus. Once cell senses a directional cue, it can use motility proteins to move toward that cue. These two processes are distinct: immobile cells still can align molecules along a chemical gradient and cells with no stimulus can migrate in random directions (Janetopoulos et al., 2004; Song et al., 2006; Swaney et al., 2010). Finally, polarized cells maintain a single “front” and “back” at designated locations in order to move processively. Again, this process is not dependent on the two others: polarized cells can migrate randomly, unpolarized cells can exhibit chemotactic behavior, and cells can maintain a polarized state without moving (Janetopoulos et al., 2004; Tang et al., 2008; Wang et al., 2014; Park et al., 2017). Because the mechanisms of polarity are less fleshed out than the other two, this review will focus on directional sensing and motility exclusively.

*Dictyostelium discoideum* undergo directed migration in the presence of chemical, mechanical, and electrical gradients, which makes it an ideal model organism for studying cell migration (Van Haastert and Devreotes, 2004; Annesley and Fisher, 2009; Artemenko et al., 2014; Nichols et al., 2015; Devreotes et al., 2017). Compared to mammalian cells, *Dictyostelium* are easy to maintain, genetically manipulate, and are amenable to large-scale genetic and pharmaceutical screens. Moreover, nearly all the signal molecules are conserved between *Dictyostelium* and mammals. Historically, *Dictyostelium* has been used to identify and study regulators of directional migration. Many of these molecules are now known, but we only have a limited understanding of how they function together as a network. *Dictyostelium* has emerged as a platform for designing and testing tools which can acutely change properties of this network. This approach, in combination with quantitative analysis and computational modeling, has allowed investigators to dissect the structure of the directed migration pathway.

### Building a Model of Directed Migration

**FIGURE 1:**
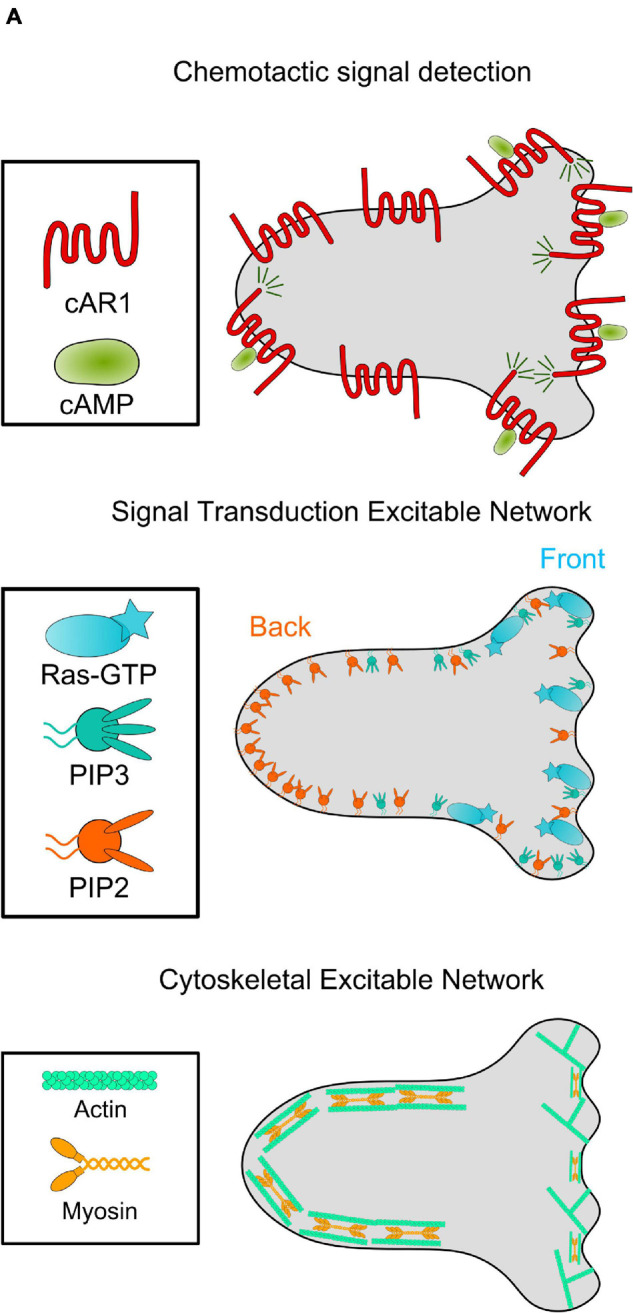
The chemotaxis network in *Dictyostelium*. Diagram of the connected networks controlling *Dictyostelium* cyclic AMP (cAMP) chemotaxis. Top: the uniformly distributed G-Protein Coupled Receptor cAR1 binds to cAMP, leading to motion up the concentration gradient. Middle: cAMP-cAR1 interactions locally increase concentration of activated Ras and membrane lipid PI(3,4,5)P3 (PIP3), among other molecules. These molecules form the cell “front” and are mutually exclusive with localization of cell “back” molecules like PI(4,5)P2 (PIP2). Together, these signaling molecules form the Signal Transduction Excitable Network. Bottom: Active Ras enrichment leads to an increase in actin polymerization at the cell front. Meanwhile, cell back signaling molecules activate the contraction of an actomyosin network. This Cytoskeletal Excitable Network powers motility.

Directed migration can be broken into three steps: chemical inputs, signal transduction, and cytoskeletal response. Ligands like Cyclic AMP (cAMP) bind to membrane-bound receptors, eliciting downstream signaling transduction events involving Ras GTPases, phosphoinositide lipids, lipid kinases/phosphatases and protein kinases/phosphatases. These molecules then modulate the actomyosin cytoskeleton network that drives cell movement (Figure 1; Devreotes et al., 2017).

**FIGURE 2:**
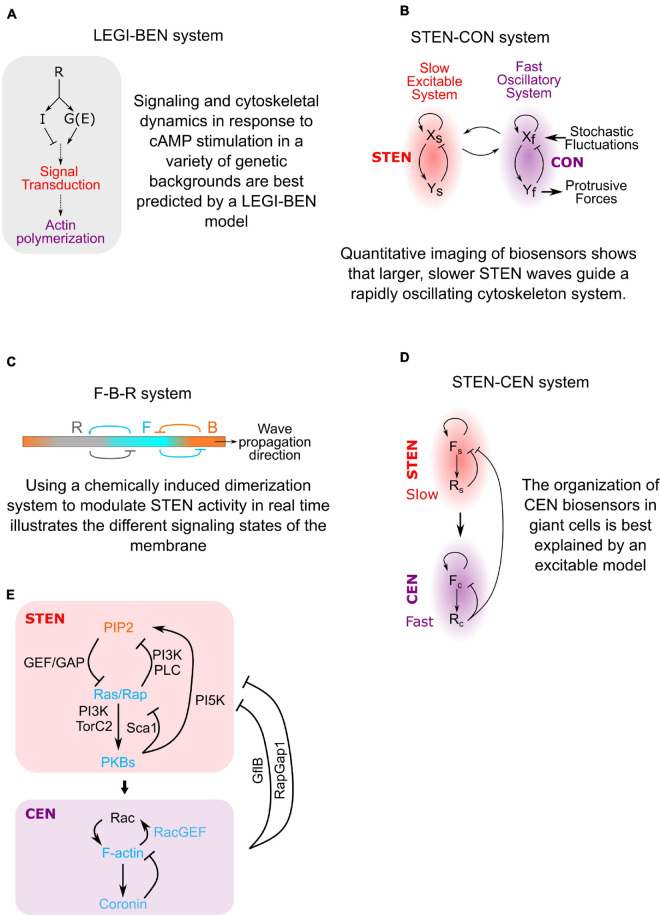
The evolution of directed migration models. **(A)** By monitoring how signaling and cytoskeletal dynamics change during cAMP stimulation in different genetic backgrounds we were able to build a model of gradient-sensing. The cell has two responses to Receptor (R) activation: locally, heterotrimeric G-proteins (G) create an Excitor (E) of signal transduction excitation. Globally, the cell produces an Inhibitor (I) which lowers the probability of excitation. This Local Excitation and Global Inhibition (LEGI) of a Biased Excitable Network (BEN) explains how cells moves in the correct direction even in shallow gradients. **(B)** Imaging of signaling biosensors independent of cytoskeletal proteins revealed excitable behavior. In the absence of signaling activity, cytoskeletal biosensors rapidly oscillate at small membrane patches. The Cytoskeletal Oscillatory Network (CON) is only able to form large, sustained protrusions when driven by the Signal Transduction Excitable Network (STEN). **(C)** Using data from chemically induced dimerization perturbations, a model of how cell front and back activities are segregated was created. The membrane sits in a resting state equivalent to the cell back state (B). STEN excitation converts a patch of the membrane to a front state (F). This F state inhibits the B state but also gradually creates its own inhibitor that converts F-state membrane to a refractory state (R). This explains how activities like Ras activation and actin polymerization propagates across cell membranes but is transient at any single location. **(D)** Biosensor dynamics show that the cytoskeletal network also has excitable properties. The Cytoskeletal Excitable Network (CEN) is coupled to the STEN but works on a faster timescale, explaining the structure of STEN-CEN waves on the bottom membrane of giant cells. **(E)** Diagram of the STEN-CEN network sketched out in panel **(D)** with specific molecules added.

In order to understand directed migration, several groups have developed computational models of the biochemical networks underlying the process (Zigmond, 1974; Mato et al., 1975; Tranquillo et al., 1988; Meinhardt, 1999; Arrieumerlou and Meyer, 2005; Levine et al., 2006; Hecht et al., 2010; Neilson et al., 2011; Takeda, 2012; Tang et al., 2014). To account for the rapid response to chemoattractant and for directional movement, the Iglesias and Devreotes labs developed a model where chemoattractant stimulation leads to Local Excitation and Global Inhibition (LEGI) (Figure 2A; Meinhardt and Gierer, 2000; Xiong et al., 2010; Tang et al., 2014). The LEGI concept explains why cells can respond directionally in shallow gradients and adapt to global concentration changes: chemical stimulation produces both an excitor and an inhibitor. The excitor is fast and localized, while the inhibitor is slow and global, ensuring that excitor is higher than inhibitor only at the high end of a gradient and lower than the inhibitor at the back. The concentration of the excitor then biases the probability of spontaneous signal transduction events such that the cell forms many more protrusions toward a stimulus on average. This LEGI framework helped explain many of the dynamic signaling changes in response to chemoattractants, but it did not detail how these changes helped cells form and control protrusions using the actin cytoskeleton.

In order to explain the coordination between signal transduction and cytoskeletal networks during cell migration, it has been proposed that these systems are directly coupled (Figures 2B-D; Vicker, 2002; Gerisch et al., 2012; Huang et al., 2013; Van Haastert et al., 2017). Measurements of signaling protein dynamics in *Dictyostelium* suggested that the signal transduction network had excitable properties similar to an action potential (Nishikawa et al., 2014; Tang et al., 2014). This **S**ignal **T**ransduction **E**xcitable **N**etwork (STEN) exhibits classic characteristics of excitability, including wave propagation, refractoriness and maximal response to suprathreshold stimuli (Xiong et al., 2010; Nishikawa et al., 2014; Tang et al., 2014). This led to the discovery that STEN organizes the activity of a rapidly oscillating cytoskeletal network to localize and shape cell protrusions (Figure 2B).

Using chemically induced dimerization to acutely change STEN activity in migrating cells, it was shown how the STEN segregates the front and back of cells: At rest, the cell membrane is dominated by phosphatidylinositol 3,4-bisphosphate[PI(3,4)P2] and phosphatidylinositol 4,5-bisphosphate[PI(4,5)P2], representing an inactive B (“Back”) state (Miao et al., 2017). These signaling molecules discourage actin polymerization and activate actomyosin contraction (Weiner et al., 2002; Wong et al., 2005; Papakonstanti et al., 2007). When extracellular signals or internal noise raise STEN activity above a threshold, it converts the local membrane to a F (“Front”) state by activating Ras/Rap proteins (Kae et al., 2004; Sasaki et al., 2004; Bolourani et al., 2006; Cai et al., 2010; Gerisch et al., 2011; Kortholt et al., 2011, 2013). These signaling proteins activate actin polymerization, a hallmark of cell fronts. The excitable behavior of STEN relies on feedback loops within the network: Ras/Rap positive feedback loops on short timescales lead to rapid activation and propagation of the F state. Longer-term negative feedback generated by front molecules places recently activated parts of the membrane into a refractory (R) state which cannot immediately fire again (Miao et al., 2017; Figure 2C).

Finally, observing the shape and kinetics of cytoskeletal activity markers after altering various regulators of signaling and cytoskeletal activity demonstrated that the cytoskeletal network displayed many of the same hallmarks of excitability as the STEN (Figure 2D; Miao et al., 2019). The **C**ytoskeletal **E**xcitable **N**etwork (CEN) model explains the organization of cytoskeletal activity biosensors relative to STEN waves on the bottom membrane of cells. The STEN-CEN model can predict signal transduction and cytoskeletal behavior in other cells, such as mammalian breast cancer cells (Xiong et al., 2016; Zhan et al., 2020).

To develop an understanding of the directed migration network, it was vital to image quantitative changes in activation and localization of dozens of molecules inside cells (Figure 2). While biochemical approaches to studying cell migration can be incredibly revealing, they nearly always eliminate spatial information and average differences across a heterogenous cell population. Comparing two fixed cell populations using immunofluorescence also has downsides: because many of the activities discussed here are variable between cells, it can be difficult to distinguish between real biological differences and noise. Imaging cellular processes in living cells circumvents these drawbacks and likely provides the most physiologically relevant information. However, studying activity changes in individual cells over time has many experimental hurdles: how can we measure changes in protein activity without damaging cells? How can we display and measure changes over time reliably? And most importantly, how can we make chemical perturbations to living cells in real time? By reviewing methods and best practices to measure and change the activity of molecules involved in cell migration, this review will provide a resource for others endeavoring to dissect complex and dynamic biological processes.

## Monitoring and Altering Migration Activities

### Reporting Activities in Living Cells in Real-Time

The dynamic localization of proteins within the cell contains important information about the organization and dynamics of the STEN and CEN networks. Many proteins localize toward a stimulus in the cell front or away from a stimulus in the cell back (Li et al., 2020). Using fluorescent fusion proteins that characterize the front or back (Table 1), we can determine the signaling state of the cell in response to a variety of perturbations. It is important to understand that while fluorescent fusion proteins indicate where a protein is, they do not report on the catalytic activity of the protein itself. For proteins that do not change activity by relocating, other types of sensors must be developed.

**TABLE 1:** Examples of endogenous proteins which can serve as fluorescent biomarkers of the cell front and back due to their well-characterized localization.

Components	Location	References
**FRONT**		
CRAC (cytosolic regulator of adenylyl cyclase)	Leading edge of migrating cell	Parent et al., 1998
Phg2	Enriched at the membrane of cell front in migrating cells, but also present in cytosol	Gebbie et al., 2004
PKBA/PKBR1	Leading edge of the migrating cells. At rest, PKBR1 is found on membrane by myristoylation, while PKBA is in cytosol	Meili et al., 1999, 2000
RacGEF1	Localized to F-actin polymerization region, mainly along the anterior cortical area and at the posterior of chemotaxing cell	Park et al., 2004
**BACK**		
PTEN	Membrane and cytosolic distribution, localized at the rear of the migrating cells. Precent on membrane in Latrunculin A treated cells	Iijima and Devreotes, 2002
ACA (adenylyl cyclase)	Back of chemotaxing cells	Kriebel et al., 2003
PAKa	The posterior cell body	Chung and Firtel, 1999
Cortexillin I	The trailing edge of migrating cells	Cha and Jeon, 2011

#### Using Biosensors to Measure Signal Transduction Excitable Network-Cytoskeletal Excitable Network Activity

To understand changes in directed migration network dynamics, it is important to measure activities, not just localizations. This is because there is often not a direct correlation between activity and localization: for example, Ras GTPase localizes to the entire cell periphery, but activated Ras (Ras-GTP) only localizes to the front of cells (Sasaki et al., 2004). Many genetically encoded fluorescent or bioluminescent sensors have been developed to observe the spatial and temporal dynamics of signaling molecules in cells. Biosensors consists of two parts: one that recognizes the analyte (signal molecules) and a reporter (fluorescent or bioluminescent proteins) (Okumoto et al., 2012). For example, the biosensor RBD (Ras Binding Domain) (Table 2) uses a protein domain that binds Ras-GTP conjugated to a fluorescent protein in order to detect activated Ras *in vivo* (Taylor et al., 2001). Biosensors can also be used to localize molecules that cannot be detected by standard fluorescence techniques. For example, the PH domain of the protein Crac (Table 1) binds to the membrane lipid PI(3,4,5)P3 and can be used to monitor PIP3 localization in the cell (Dormann et al., 2002).

**TABLE 2:** Examples of well-characterized biosensors for important activities in the celll migration network.

Binding targets	Biosensor	*In vivo* location	Protein domain	References
Actin filament (F-actin)	LimE△coil	Protrusions of cell front	A LIM-domain containing protein DdLimE deleted c-terminal coiled-coil domain	Bretschneider et al., 2004
	Lifeact	filamentous actin in whole cell	A 17-amino-acid peptide	Riedl et al., 2008
Ras-GTP	RBD (Ras binding domain)	Active patches on protrusions and macropinocytic cups	Ras binding domain (RBD) of c-Raf-1 (amino acids 51–131)	Chiu et al., 2002
Rap1-GTP	RBDRalGDS	Protrusions of cell fronts	Ras binding domain (RBD) of RalGDS	Bivona et al., 2004
PI(3,4)P2	CynA	Trailing edge and macropinocytic cups	PH domain-containing proteins PH21 that binds PI(3,4)P2	Swaney et al., 2015
PI(4,5)P2	PH-PLCδ1	lamellipodium, base of the endocytic invagination	PH domain of PLCδ1that bind PI45P2. But binds to IP3 ∼ 20 fold more tightly than PI(4,5)P2	Wills et al., 2018
PIP3	PHcrac	Protrusions of cell front, macropinocytic cups	PH (pleckstrin homology) domain of cytosolic regulator of adenylyl cyclase (CRAC)	Huang et al., 2003
Rac-GDP	Coronin CRIB motif	Protrusions of cell fronts, colocalized with polymerizing F-actin	Cdc42- and Rac-interactive binding motif (CRIB) of coronin	Swaminathan et al., 2014
Rac1-GTP	PAK-PBD	Rac1-GTP is throughout the phagocytic cup	The Rac/Cdc42 (p21) binding domain (PBD) of the human p21 activated kinase 1 protein (PAK)	Benard et al., 1999

One important note of caution is that biosensor localization does not linearly correlate with activity. Depending on expression level and affinity, only a fraction of the biosensor is bound to the analyte. For example, there are two main populations of RBD within a *Dictyostelium* cell: localized to the front region of the membrane and in the cytoplasm. The cytoplasmic pool of RBD contains unbound biosensor and does not indicate Ras activation. Therefore, the specific biology of the analyte must be considered when interpreting data from biosensor experiments.

#### Using Electrofused Cells to Image Signal Transduction Excitable Network-Cytoskeletal Excitable Network Waves on the Bottom Membrane

**FIGURE 3:**
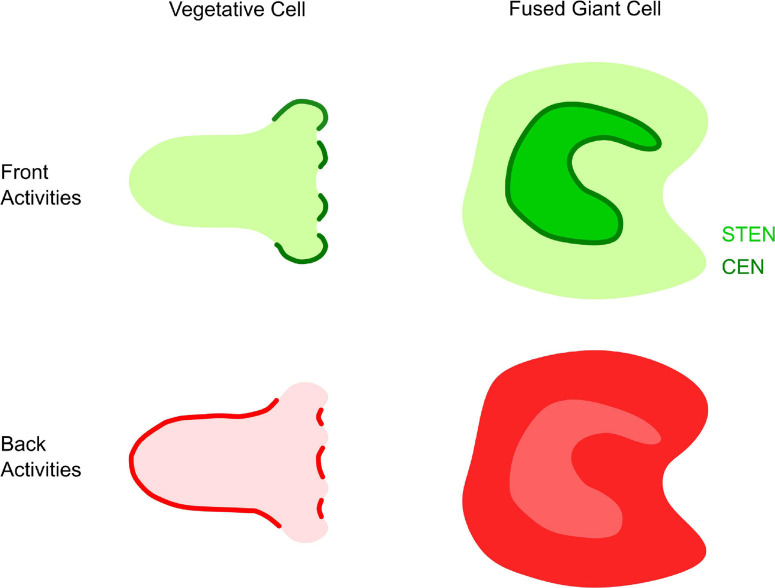
Front and back organization in *Dictyostelium*. Illustration of “front” and “back” activities in the middle focal plane of a single vegetative cells and the bottom membrane of giant cells. Left: in single cells, front (green) activities occur at protrusions or macropinocytic cups, while back (red) activities display complementary patterns at the trailing edge of macropinocytic cups or the cell. Right: in giant cells, front activities (green) propagate as cortical waves. STEN (light green) activities are enclosed by CEN (dark green). Back activities (dark red) are excluded from the area of front activity, creating “shadow waves” of low intensity when imaging back biosensors (light red).

In single cells, it is hard to dissect the temporal order of signals in same network because signaling events seems to all initiate spontaneously and stochastically. One reason for this is the small dimensions of a cell: protrusions formed on the cell periphery can quickly travel vertically out of the imaging plane (Figure 3). Meanwhile, signaling events starting on the center of the bottom membrane can quickly reach the periphery and go out of view. To gain insight in the spatiotemporal regulation of the chemotactic network, Gerisch and colleagues fused multiple *Dictyostelium* cells using electric pulses and described the generation and propagation of self-organized actin waves on the substrate-attached surface (Gerisch et al., 2013; Gerhardt et al., 2014). Subsequently, giant cells generated by electropulse-induced cell fusion have become a tool for studying the spatial organization of STEN and CEN molecules (Figure 3). The giant surface is an ideal platform for observing the propagation of signaling events: Ras, PIP3 and actin polymerization propagate along the surface in coordinated waves (Schroth-Diez et al., 2009; Arai et al., 2010; Shibata et al., 2012; Taniguchi et al., 2013; Gerhardt et al., 2014). Usually, active F state molecules like Ras-GTP, PIP3 form waves that appear to be surrounded by CEN waves (actin) (Miao et al., 2019). Outside the waves are B state molecules such as PTEN. The spatial “phase shift” between waves of the various sensors reveal the organization and kinetics of STEN and CEN. STEN biosensors show a one-peak wave with Rap activity leading Ras activity, in front of PIP3, and followed by PKB. CEN waves span the STEN region but also have an initial peak and a weaker trailing peak. This two peak structure is best explained by a model where the timescale of CEN activation and inhibition is much faster than STEN (Miao et al., 2019).

#### Altering Protein Activity in Living Cells

The directed migration network contains hundreds of molecules which are linked in an intricate series of feedback loops. Because of this, assessing the role of one individual molecule is difficult: adaptive changes in the level or activity of other proteins can compensate for individual deletions, leading to unexpected phenotypes. Because of this, it is critical to observe how changes in protein activity affect the network immediately. For example, transiently raising Ras activity has different effects on cell morphology than expressing constitutively activated Ras (Miao et al., 2017; Edwards et al., 2018). While this section will focus on newer synthetic methods to alter cell chemistry, some older tools which remain in use today will be briefly noted.

#### Stimulation of the Directed Migration Network

For decades, researchers have been using the natural response of *Dictyostelium* to chemical signals to alter the directed migration pathway in real time. Cyclic AMP (cAMP) activates a chemotactic response in *Dictyostelium* by binding the GPCR cAR1, leading to elevated Ras activation and actin polymerization at the high end of the gradient. The localization and dynamics of a molecule during cAMP stimulation contain important information about that molecule’s position in the directed migration pathway (Devreotes et al., 2017). While representing the true chemotactic response, this approach has a few disadvantages for unraveling network architecture: it alters the activity of many proteins at once, making specific studies more difficult. Cells developed to respond to cAMP are also more polarized complicating direct comparison to the undeveloped cells. However, cAMP activation is fast (2-3 s), robust over many mutant lines, tunable at low concentrations, and does not require any genetic perturbations. This flexibility and accessibility make it a useful tool to place individual molecules within the chemotaxis pathway. Because it requires no molecular biology tools, cAR1 stimulation can even be combined with other techniques like chemical dimerization (Miao et al., 2017) to measure how acute changes in other molecules affect the pathway’s response to stimuli. Finally, if cAMP is not feasible, many orthogonal methods to activate the directed migration pathway exist: chemicals like folic acid which act on a different receptor, mechanical forces like shear flow, and electrical fields (Zhao et al., 2002; Décave et al., 2003; Artemenko et al., 2016).

#### Pharmacological Perturbations

Many *Dictyostelium* proteins involved in directed migration can be controlled with the use of small molecule inhibitors. Commonly used inhibitors like the actin depolymerizing agent latrunculin the PI3K inhibitor LY294002, the Myosin inhibitor blebbistatin, and the TORC2 inhibitor PP242 are effective in altering STEN and CEN activities in *Dictyostelium* (Gerisch et al., 2004; Shu et al., 2005; Loovers et al., 2006; Cai et al., 2010). There are significant advantages to pharmacological approaches: they are fast, potentially address only a single node in the network, and do not require genetic manipulations. The main drawback to these techniques is availability: most newly developed small molecule inhibitors are optimized to function in human cells for medical applications and may not function well in *Dictyostelium*. Additionally, inhibitors may have significant off-target effects which confound results.

#### Chemically Induced Dimerization

**FIGURE 4:**
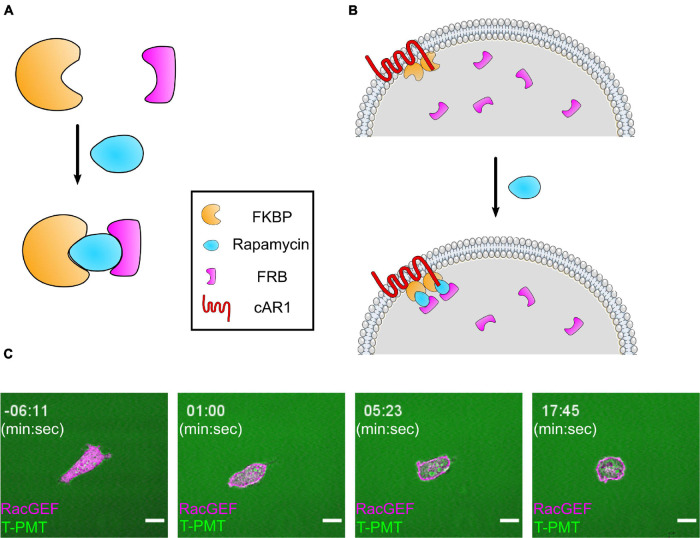
The FKBP/FRB system and its application in cells. **(A)** Diagram of FKBP/FRB system. FKBP and FRB form a heterodimer in the presence of rapamycin. **(B)** Illustration of an experimental setup for recruiting a protein to the membrane with the FKBP/FRB system in *Dictyostelium*. 2 FKBP domains (FX2) are anchored to membrane by cAR1, and the actuator attached to an FRB domain is diffusing in the cytosol. When rapamycin is added, the actuator is recruited to membrane by binding to FKBP. **(C)** Scanning confocal imaging of an AX3 *Dictyostelium* cell expressing a CID system designed to increase RacB and Rac1A activity. The cell is expressing cAR1-FKBP-FKBP (unlabeled) and mCherry-FRB-RacGEF1_Δ*N*_, an activator of Rac1 fused to an FRB domain. After rapamycin addition, RacGEF1_Δ*N*_ is recruited to the membrane. Consistent with previous reports, the cell became very round with small, short-lived protrusions. Scale bars = 10 μm. *t* = 00:00 indicates rapamycin addition.

**C**hemically **I**nduced **D**imerization (CID) is a flexible method for recruiting proteins to specific locations in the cell. In CID, a protein of interest (an “actuator”) is fused to a protein domain which, upon the addition of a chemical agent, will dimerize with another protein domain fused to an organelle (an “anchor”) (Figure 4). While there a few different versions of this system (Fegan et al., 2010), this section will focus on the well-established FKBP-FRB system. The FKBP-FRB system takes advantage of the ability of **FK**506 **B**inding **P**rotein (FKBP) to heterodimerize with a domain of the signaling protein mTOR (the helpfully named **F**KBP-**R**apamycin **B**inding domain, or FRB) in the presence of the small molecule Rapamycin (Derose et al., 2013). CID is capable of targeting protein activity to certain intracellular regions and is specific to the protein of interest. The FKBP-FRB system also acts within seconds of rapamycin addition and does not require any specialized equipment. These properties make CID an ideal tool to study a complex, dynamic network like the chemotaxis pathway.

The CID system allows researchers to separate how changes in different parts of the directed migration network affect cell function. For example, a truncated, membrane-recruitable RacGEF1 can be used to increase actin polymerization across the cell membrane (Figure 4C and Supplementary Movie 1). While cAMP stimulation leads to actin polymerization through a signaling cascade, this perturbation directly activates the actin nucleator WASP. Similar to cAMP stimulation and STEN activation, RacGEF1 membrane recruitment causes cells to flatten out as actin polymerizes in all directions (Figure 4C and Supplementary Movie 1) (Miao et al., 2019). However, unlike upstream perturbations, direct upregulation of actin polymerization leads to a large decrease in Ras activation (Miao et al., 2019). This negative feedback from CEN to STEN was only uncovered by choosing a specific downstream entry point into the chemotaxis pathway. When designing FKBP-FRB experiments in *Dictyostelium* like this one, there are many important considerations in design and execution, which will be discussed below. For a summary of these considerations, see Quick Start Guide 1.

**QUICK START GUIDE 1:**
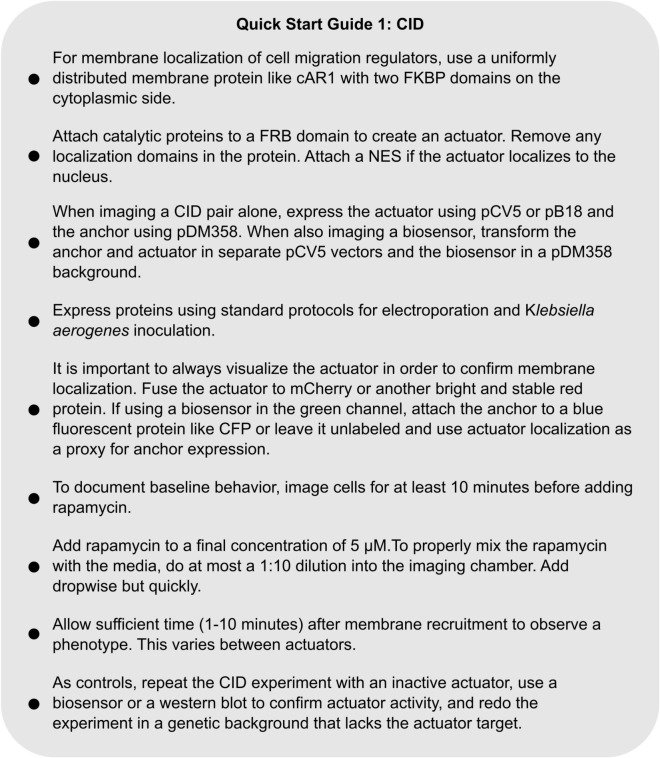
Instructions for designing and carrying out a chemically induced dimerization experiment in *Dictyostelium*.

##### Organelle Anchor Design

The anchor fusion protein determines where a protein of interest localizes within a cell once rapamycin is added. In order to function correctly, it must be stable, highly expressed, and uniformly distributed at the organelle of choice. Because most regulation of directed migration occurs at the membrane or close to it at the cortex, this guide will focus on anchors to recruit proteins to the inner side of the plasma membrane. For a list of other organelle localization strategies, see Derose et al. (2013). Designing a plasma membrane anchor is simple: the targeting sequence or protein must localize uniformly at the plasma membrane and not interfere with cell function. To ensure that the actuator localizes to the membrane instead of the reverse, the anchor must also have slow turnover rate within the plasma membrane. For specific details of recommended anchors, see the Supplementary Methods.

##### Actuator Design

The design of actuators is more involved and varied than anchors. When adapting a protein for use in a CID system, it is important to take its specific biology into account. To illustrate, consider a system designed to transiently raise Ras activity. Because Ras targets involved in cell migration are primarily at the membrane, a constitutively active FRB-tagged RasC mutant (RasC_*Q*62*L*_) which could be recruited to the membrane was created. To prevent actuator expression from affecting the cell prior to rapamycin addition, the membrane localization domain (a C-terminal CAAX) was deleted (Miao et al., 2017). When this FRB-RasC_*Q*62*L*Δ*CAAX*_ was expressed along with a Myr-2XFKBP anchor, robust membrane localization of the actuator was observed only after rapamycin addition. FRB-RasC_*Q*62*L*Δ *CAAX*_ membrane localization coincided with large increases in PIP3 levels, actin polymerization, and protrusion size. A similar approach can be taken to many classes of molecules, including kinases, phosphatases, and GEFs (Miao et al., 2019). As a general guideline, actuators must be catalytic (as opposed to structural), specific to a process of interest, and have low activity when not localized to the correct region of the cell.

##### Expression Guidelines – Vectors and Fluorescent Proteins

The primary consideration for expressing CID components is actuator abundance. In previous experiments, transforming the actuator in a high copy number vector was required to observe a phenotype after recruitment (Miao et al., 2017, 2019). When imaging cells in a CID experiment, it is critical to confirm that the actuator is expressed at high levels and localizes to the membrane after rapamycin addition. Therefore, the actuator should always be fused to a bright fluorescent protein. While imaging the membrane anchor is helpful for confirming expression, it is often more useful to leave the anchor unlabeled and also express a fluorescent biosensor. For example, by imaging GFP-conjugated PIP3 and actin polymerization biosensors before and after recruitment of mCherry-FRB-RasC_*Q*62*L*Δ*CAAX*_, the Devreotes Lab showed that transient increases in Ras activation led to larger fronts of Actin and PIP3 (Miao et al., 2017). Refer to the Supplementary Methods for more information.

##### Experimental Practice and Controls

To confirm that a CID system is working as intended, it is important to show that phenotypic changes are due to the activity of the localized protein rather than some effect of rapamycin addition or an off-target effect. Repeat the CID experiment with an inactive version of the activator, like a catalytic domain mutant. Additionally, confirm that the actuator is acting on its target: For example, membrane localization of a PI(4,5)P2 biosensor should drop after recruiting a PI(4,5) phosphatase (Miao et al., 2017). This can also be done by measuring bulk differences in the levels of a target protein using western blot. However heterogeneous expression of the CID system makes it difficult to observe population-level changes. Finally, confirm that the actuator is affecting the desired pathway by repeating the CID experiment in a knockout or knockdown strain of the intended target protein. For specific guidelines for setting up and performing a CID experiment, see the Supplementary Methods.

#### Optogenetic Control of Cell Activity

**FIGURE 5:**
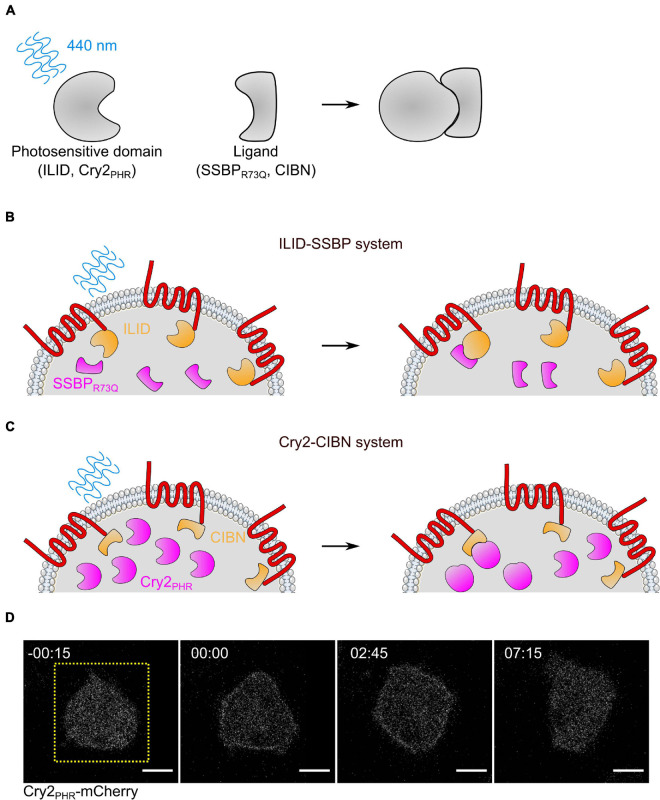
Optogenetic systems for controlling protein localization in *Dictyostelium.* **(A)** Diagram of blue light dimerization systems. In the dark, a photosensitive protein (iLID or Cry2_*PHR*_) has low affinity for its ligand (SSBP_*R73Q*_ or CIBN, respectively). When exposed to 450 nm light, a conformational change in the photosensitive domain allows it to bind to the ligand with affinity. **(B,C)** Diagram of iLID-based (B) and Cry2-based (C) systems for recruiting proteins to the membrane in *Dictyostelium*. The actuator (magenta) is recruited to the uniformly distributed membrane anchor (orange) only at the area exposed to blue light. Note that in the iLID system, the photosensitive protein is attached to the membrane anchor, while in the Cry2 system, it is attached to the cytosolic actuator. **(D)** Scanning confocal imaging of Cry2_*PHR*_ membrane recruitment in AX3 *Dictyostelium* cells. Cells are also expressing an unlabeled membrane anchor, cAR1-CIBN. Yellow dashed square indicates region illuminated with 488 nm light. Scale bars = 5 μm. *t* = 00:00 indicates blue light stimulation.

In cell biology, optogenetics is a tool for controlling the location and activity of proteins within living cells (Figure 5; Toettcher et al., 2011). Optogenetics takes advantage of protein domains that change conformation when exposed to a specific wavelength of light. This conformational change exposes a binding domain or separates protein domains (Kennedy et al., 2010; Strickland et al., 2012; Wang et al., 2016; Van Haren et al., 2018). In conjunction with a patterned illumination system like a Digital Micromirror Device (DMD) or a scanning confocal, optogenetic systems allow for temporal control of protein activity at the subcellular level. This review will focus two separate optogenetic systems which are relatively user-friendly: Cry2_*PHR*_-CIBN and iLID-SSBP (Kennedy et al., 2010; Guntas et al., 2015). Like CID, these systems can be used to recruit cell migration regulators to the cell membrane by attaching one domain to a uniform membrane protein and one to a catalytic domain. This has some distinct advantages over CID experiments, including subcellular control, reversibility, and the ability to perform many sequential experiments on one dish. However, optogenetics requires more specialized equipment than CID experiments and both systems are activated by 488 nm light (maximally at 440 nm), making it impossible to image green fluorescent proteins. For a summary of optogenetics instructions, see Quick Start Guide 2.

**QUICK START GUIDE 2:**
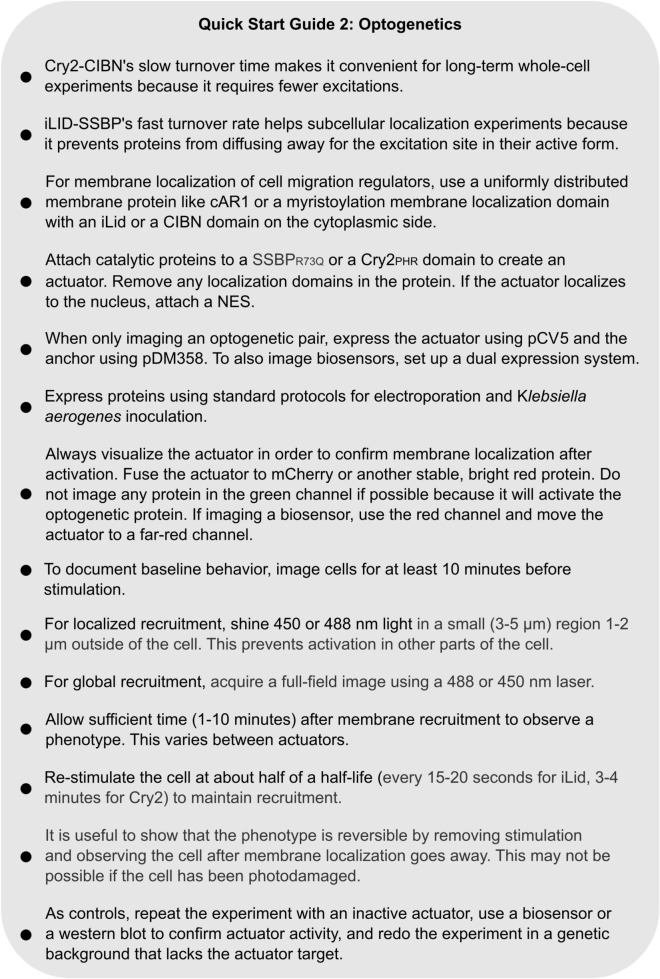
Instructions for designing and carrying out an optogenetic experiment with Cry2-CIBN or an iLID system in *Dictyostelium*.

##### Cry2-CIBN vs. iLID-SSBP

The Cry2_*PHR*_ domain, based on a plant cryptochrome, binds to CIBN domains in the presence of 450 nm light (Kennedy et al., 2010). Similarly, iLID is a modified LOV domain from *Avena sativa* that binds to a small, engineered peptide – SSBP – when exposed to blue light (Guntas et al., 2015). The primary difference is turnover time: the Cry2-CIBN interaction has a half-life of 6 min while iLID-SSBP has a half-life of 30 seconds. The short half-life of iLID makes it ideal for subcellular localization because stimulated protein cannot diffuse far from the excitation site. In Cry2 systems, the activated protein can diffuse far away from the light source while remaining active. However, Cry2 requires less periodic reactivation to maintain recruitment, making it easier to work with for whole-cell experiments. In *Dictyostelium*, Cry2 rapidly localizes to the plasma membrane of cells expressing a CIBN membrane anchor, and remains localized minutes later (Figure 5D and Supplementary Movie 2). Which protein is more suitable for an experiment depends on the specific biology of the system to be perturbed. For more information about designing optogenetic constructs and performing optogenetic experiments, see the Supplementary Methods.

## Analyzing Signaling Changes

There are two primary experimental readouts to quantify after altering cell behavior: cell morphology and biosensor organization. Both properties provide important context that the other does not. For example, it has recently been observed that there are at least two mechanisms which lead to an increase in cell area: increased actin polymerization and increased cell adhesion (Thomas Lampert et al., 2017). Without imaging biosensors of actin polymerization, it would be difficult to determine why a particular actuator leads to cell flattening. The section below will describe how to quantify and display changes in cell morphology and biosensors before and after altering protein activities.

### Experimental Guidelines for Analyzing Cells Quantitatively

One of the most important parts of an image analysis pipeline is the initial design of the imaging experiment. There are many problems that are very difficult and time-consuming to fix when processing images which can be avoided when acquiring the data. For example, if two cells touch each other, simple segmentation algorithms often join them into one object, altering the cell area and shape. There are solutions to this problem (e.g., watershed algorithms, Gamarra et al., 2019), but they often need to be tuned dynamically between time points and cells. It is much simpler to plate cells at a low enough density to make two labeled cells touching unlikely. For specific recommendations for plating and imaging cells, see the Supplementary Methods and Quick Start Guide 3.

**QUICK START GUIDE 3:**
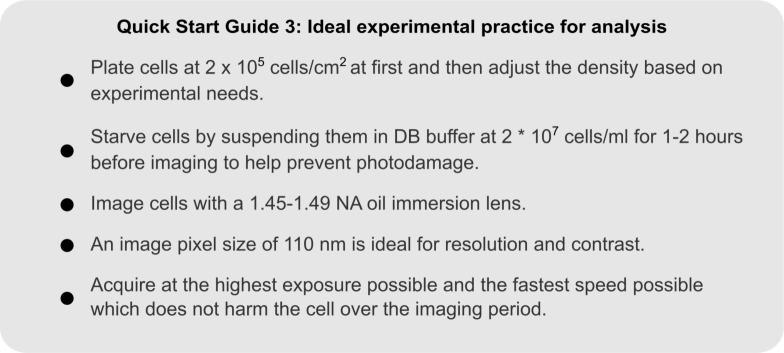
Guidelines for optimizing *Dictyostelium* imaging conditions, with the goal of quantitatively analyzing the output.

### Analyzing Cell Behavior and Morphology

Changing the activity or levels of cell migration regulators can effect cell size, shape, and speed (Devreotes et al., 2017). These properties can be quantified without specialized labels and contain important information about underlying biological changes. Cell shape and motion can be measured manually using a program like ImageJ (Schindelin et al., 2012) or automatically with ImageJ scripting or a coding platform like Matlab or Python’s OpenCV library. There are tradeoffs to both methods: identifying cells by hand can be slow and subject to bias. However, writing code that can consistently find and track cells across many different datasets can potentially be more time-consuming than simply tracing the outline of cells frame-by-frame. Some important guidelines for each approach are discussed below.

#### Manual Quantification

In a CID or optogenetic experiment, either the actuator or the anchor can be used to track cells. Transmitted light techniques like DIC or phase contrast can also be used, though it may produce slightly different results than fluorescence depending on the imaging modality. Manual tracking can quickly uncover changes in cell behavior after perturbing directed migration machinery. For example, in 2017 the Devreotes lab analyzed the motion of cells after lowering the STEN threshold by reducing PI(4,5)P2 levels. These cells exhibited one of three distinct migratory modes: amoeboid cells moved small distances and changed directions, fan-shaped cells moved large distances in a single direction, and oscillators intermittently stopped before moving in a new random direction. While these populations could be segregated based on motion alone, they also had different morphologies: amoeboid cells had consistently low areas, fan-shaped cells had larger areas and a clear long axis, and oscillators showed periodic changes in area over a movement cycle (Miao et al., 2017). The observation that altering STEN properties dramatically alters the morphology of a migrating cell suggests that varying STEN and CEN activity could be partially responsible for the diversity of migratory behavior in nature. For more information on how to measure cell shape and track cells, refer to the Supplementary Methods and Quick Start Guide 4.

**QUICK START GUIDE 4:**
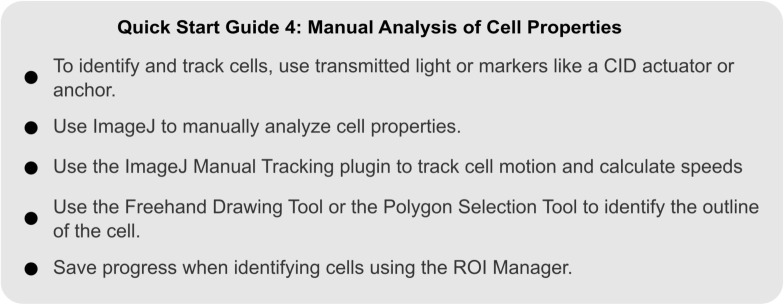
Instructions for manually analyzing cell shape and motion in migrating *Dictyostelium* cells.

#### Automatic Quantification

There are many pre-written programs designed to identify and track cells in motion (Emami et al., 2021), although they are not optimized for *Dictyostelium*. For tracking hundreds of cells adapting one of these approaches may be effective. For smaller sample sizes, manually tracking the cells in ImageJ using the Manual Tracking plugin is a simple way to skip a computationally difficult step. Then, with ImageJ, Matlab, or another analysis platform, use the actuator or anchor fluorescence to identify the shape of the cell. Refer to Supplementary Materials. Quick Start Guide 5 summarizes this segmentation protocol.

**QUICK START GUIDE 5:**
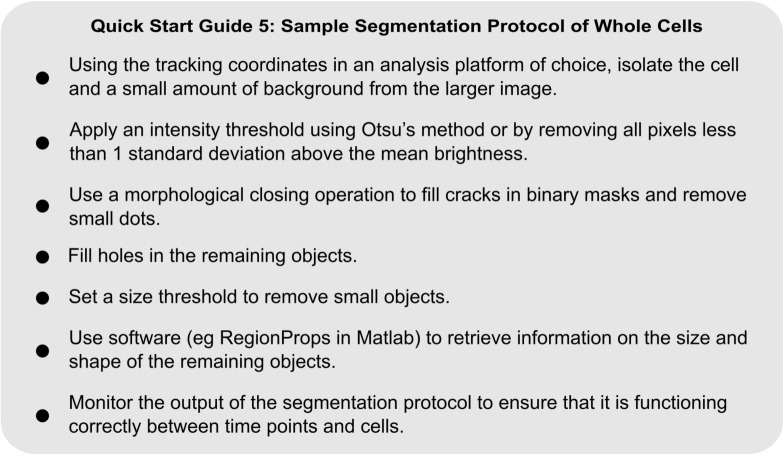
Sample protocol for the automated identification of a *Dictyostelium* cell from microscopy images.

### Quantifying Fluorescent Biosensors

Fluorescent biosensors report on the level and location, and activity of specific molecules within the cell. In the context of directed cell migration, STEN and CEN biosensors localize along the cell periphery in patches to shape protrusions and travel along the bottom membrane as waves. The properties of these patches and waves (size, brightness, speed, location) contain important information about the properties of the directed migration network. How to quantify changes in STEN and CEN by monitoring bottom membrane waves and protrusions will be discussed here below.

#### Quantifying Signal Transduction Excitable Network-Cytoskeletal Excitable Network Wave Properties

**FIGURE 6:**
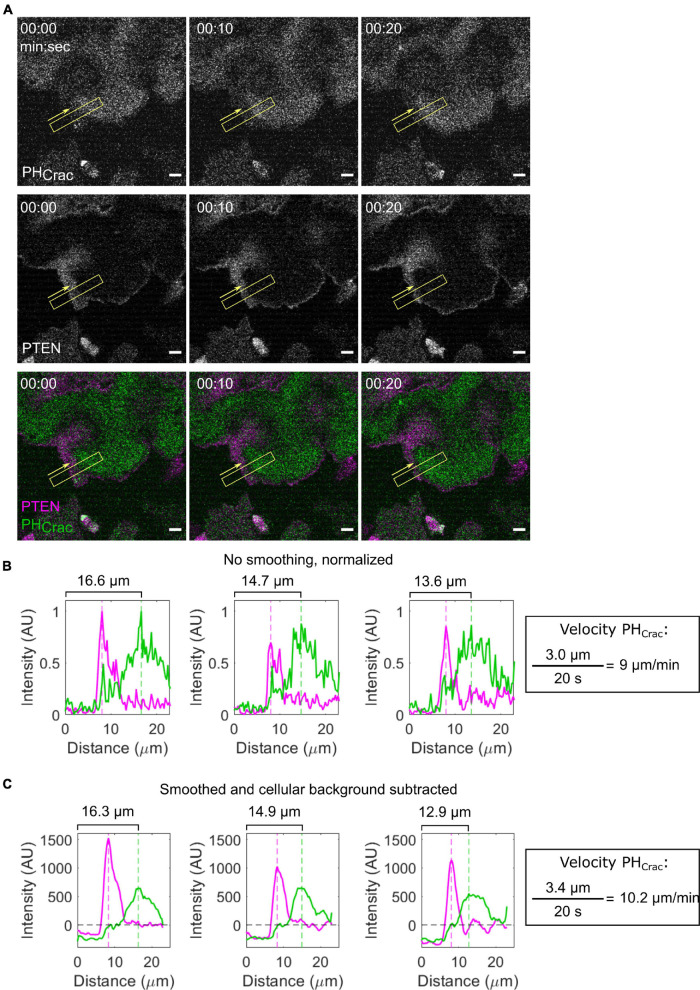
Measuring STEN wave properties. **(A)** Scanning confocal imaging of PIP3 (PH_*Crac*_) and a back protein (PTEN) in a giant AX2 *Dictyostelium* cell. Yellow boxes indicate the location and width of the intensity line scan across a membrane wave. Yellow arrows indicate the direction of the line scan. **(B,C)** Raw (B) and smoothed and background-corrected (C) Intensity line scans corresponding to the timepoints in panel **(A)**. The position of the linescan area does not change, while the peak position of PH_*CRAC*_ decreases. This change can be used to calculate the velocity of the front wave.

Signal transduction excitable network and CEN biosensors travel in waves of front activity and complementary waves of back activity on the cell bottom (Figure 6A and Supplementary Movie 3). Previously, it has been shown that the peak-to-peak distance of STEN and CEN biosensors reports on their order in the directed migration pathway (Miao et al., 2019). Additionally, their speed, size, and propagation distance correspond to the timescale, activity, and threshold of STEN and CEN. Many of these properties can be measured by hand in ImageJ: using the line tool, draw a line in the direction of wave propagation which includes the wave and cellular background on both sides. Then, the Plot Profile tool can be used to obtain the intensity of all pixels along that line for each biosensor. Increasing the width of the line causes the tool to average over more pixels, decreasing measurement noise. After subtracting cellular background, the distance between two biosensors can be calculated by the difference between the maxima of each biosensor. This intensity profile can be also used to calculate the speed of the wave between frames and the integrated intensity of the biosensor (Figures 6B,C). For example, PI(3,4,5)P3 biosensor PH_*CRAC*_ travels along the bottom membrane as a part of a front STEN wave (Figure 6A and Supplementary Movie 3). Back proteins, like PI(3,4,5)P3 phosphatase PTEN, are excluded from this same area. The speed of the STEN wave can be obtained by measuring the location of the PH_*Crac*_ maxima over time (Figures 6B,C). This speed is directly influenced by STEN and CEN activity and can be increased by lowering the STEN threshold (Miao et al., 2019). Because these waves are bright and large structures, the previously described segmentation protocols can also be used for automated identification and analysis.

#### Quantifying Signal Transduction Excitable Network-Cytoskeletal Excitable Network Events on the Cell Periphery

**FIGURE 7:**
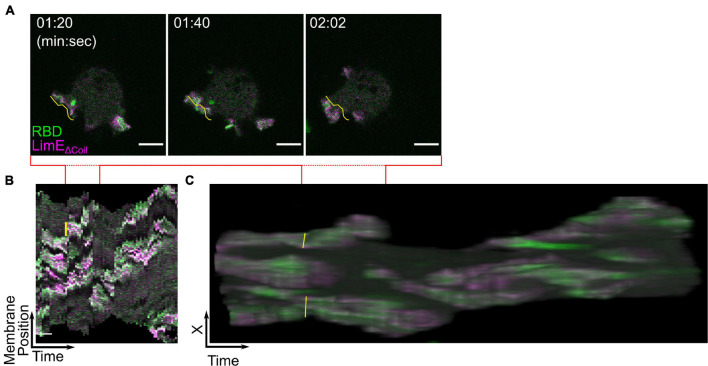
Displaying biosensors on cell protrusions. (**A–C)** Scanning confocal imaging **(A)**, membrane kymograph **(B)**, and t-stack **(C)** of Ras activation (RBD) and actin polymerization (LimE_Δ*Coil*_) in AX3 *Dictyostelium* cells. The yellow line highlights the same protrusion in all three images. Scale bars in panels **(A)** = 5 μm and **(B)** = 30 s. *t* = 00:00 indicates the start of acquisition. Red lines indicate the span of time covered by the still images in panel **(A)**.

Like the bottom membrane, the size and shape of STEN-CEN events that create protrusions on the cell periphery contain important information about network properties (Miao et al., 2017). It is possible to measure the size of cell protrusions by hand using ImageJ. However, the number of measurements required per cell may be prohibitive. For example, actin polymerization biosensor LimE_Δ*Coil*_ and Ras activity biosensor RBD colocalize at multiple protrusions along the *Dictyostelium* cell perimeter (Figure 7A and Supplementary Movie 4). Each of these protrusions undergoes dramatic shape changes between frames, making the assessment of a protrusion’s behavior over longer times difficult. To measure signaling events on cell perimeter, creating membrane kymographs and t-stacks is recommended. The basic concept of a membrane kymograph is simple: trace the cell membrane at each frame, divide the traced line into equidistant points, and then display the biosensor intensity at each point as a vertical line. Finally, stack each timepoint’s vertical line horizontally to create a single image which reports on changes in biosensor intensity on the membrane across an entire movie. A single protrusion in the original image (Figure 7A, yellow line) is simplified to small region of the kymograph (Figure 7B, yellow line) where the height indicates the arc length on the membrane and the width indicates the duration. These quantities can be obtained with single measurements in ImageJ. The code to generate such a kymograph is flexible and available upon request. T-stacks are made by taking a timelapse movie of a migrating cell and creating a 3D projection where the third axis is time (Figure 7C). When viewed in the X-T plane (e.g., looking at the edge of the cell over time), the duration, movement, and width, and length of protrusions can be seen and measured. Quick Start Guide 6 details these options.

**QUICK START GUIDE 6:**
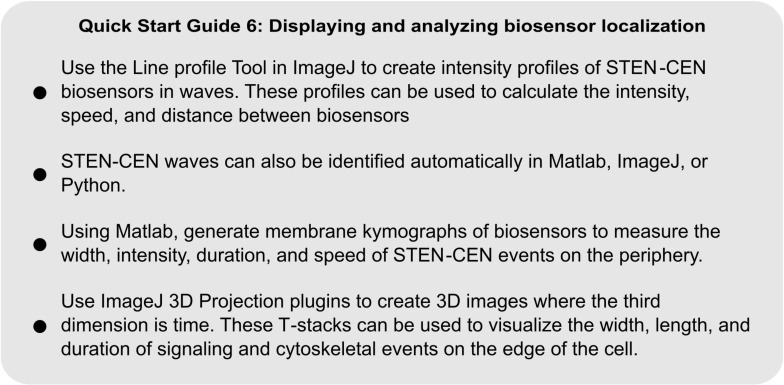
Guidelines for displaying and analyzing fluorescent biosensor dynamics on the cell membrane.

## Conclusion

### Future Applications of Chemically Induced Dimerization and Optogenetic Techniques to Study Cell Migration

In conjunction with other emerging technologies, CID and optogenetic techniques can help deepen our understanding of how cells move toward signals through difficult environments. For example, micropatterning and microfluidic techniques can subject cells to geometrical and mechanical challenges like they may encounter in a dynamic tissue (Reversat et al., 2020). Acutely altering the activity of individual molecules in a cell in this situation can teach us what is and is not sufficient for robust migration. Additionally, it is possible to use modern imaging technologies like Lattice Lightsheet Microscopy (LLSM) to get dynamic three-dimensional images of cellular structures at high resolution (Gao et al., 2012; Fritz-Laylin et al., 2017). LLSM could allow us to measure how the true structure of a protrusion or STEN-CEN wave changes in response to changes in network regulators. Finally, machine learning algorithms capable of automatically identifying cells and subcellular structures (Al-Kofahi et al., 2018) could help identify subtle changes to cell shape and activity which are not apparent in small datasets. Together, these approaches will help to develop and refine the current working model of directed migration in eukaryotes.

### Using Live Cell Perturbations to Study Other Problems in *Dictyostelium*

Many of the same techniques described here for studying cell migration can also be applied to other biological problems. For example, *Dictyostelium* are commonly used to study mechanotransduction and cytokinesis (Liu and Robinson, 2018). Recruiting a Myosin regulator to the cortex or an Aurora Kinase effector to the midzone during cytokinesis may give important quantitative insight about the network that localizes and contracts the cytokinetic ring. Additionally, the formation of multicellular slugs and fruiting bodies during the *Dictyostelium* life cycle is a popular model for multicellular organization (Williams, 2010). Changing the activity of specific molecules at particular times during development many have large effects on body shape which would not be apparent from longer-term genetic manipulations. Finally, *Dictyostelium* is an excellent model for studying micropinocytosis and phagocytosis (Vogel et al., 1980; Bloomfield et al., 2015; Dunn et al., 2018). Macropinocytosis and phagocytosis are tightly regulated in time and share many regulators with directed migration. This similarity makes them an excellent candidate for study using the tools described above. Emerging techniques in synthetic and computational biology will allow us to study old problems in *Dictyostelium* with new levels of detail and insight.

### Applying Insights From *Dictyostelium* to Other Organisms

As a model organism, *Dictyostelium* has expanded our understanding of chemotaxis. Importantly, lessons learned in *Dictyostelium* can be applied to other systems. For example, in *Dictyostelium* chemotaxis: gradient sensing, signal transduction and cell motility. These processes all require interactions with receptors on the cell surface which trigger polarized distribution of downstream effectors located at front and back. This distribution rearranges the cytoskeleton in order to move toward a gradient. The signaling network discovered in *Dictyostelium* is also highly conserved in eukaryotic cells (Artemenko et al., 2014). Because of this, and because *Dictyostelium* is easy to culture, manipulate, and image, it is a good platform for designing and testing CID and optogenetic tools for use in other cell migration models. For example, an actuator which lowers PIP2 levels was recently adapted for use in mammalian breast epithelial cells (Zhan et al., 2020). We propose using *Dictyostelium* as a system to rapidly generate hypotheses and build models which can then be broadly applied.

## Author Contributions

JK and YL wrote the manuscript, acquired the data, and made figures. PD provided conceptual guidance and edited the manuscript. All authors contributed to the article and approved the submitted version.

## Conflict of Interest

The authors declare that the research was conducted in the absence of any commercial or financial relationships that could be construed as a potential conflict of interest.

## Publisher’s Note

All claims expressed in this article are solely those of the authors and do not necessarily represent those of their affiliated organizations, or those of the publisher, the editors and the reviewers. Any product that may be evaluated in this article, or claim that may be made by its manufacturer, is not guaranteed or endorsed by the publisher.

## Supplementary Material

Supplementary Movie 1 Increasing RacB and Rac1A activity using a CID system. Scanning confocal imaging of an AX3 *Dictyostelium* cell (transmitted light, green) expressing a CID system designed to increase RacB and Rac1A activity. The cell is expressing cAR1-FKBP-FKBP (unlabeled) and mCherry-FRB-RacGEF1_Δ *N*_ (magenta), an activator of Rac1 fused to an FRB domain. After rapamycin addition, RacGEF1_Δ *N*_ is recruited to the membrane. Consistent with previous reports, the cell became very round with small, short-lived protrusions. *t* = 00:00 indicates rapamycin addition. Playback is 18 frames per second.

Supplementary Movie 2 The Cry2_*PHR*_-CIBN system in *Dictyostelium*. Scanning confocal imaging of Cry2_*PHR*_ membrane recruitment in AX3 *Dictyostelium* cells. Cells are also expressing an unlabeled membrane anchor, cAR1-CIBN. Yellow square indicates region illuminated with 488 nm light. *t* = 00:00 indicates blue light stimulation. Playback is 22 frames per second.

Supplementary Movie 3 Traveling STEN waves on the bottom of a giant cell. Scanning confocal imaging of PIP3 (PH_*Crac*_, green) and a back protein (PTEN, magenta) in a giant AX2 *Dictyostelium* cell. Playback is 2 frames per second.

Supplementary Movie 4 Signal transduction excitable network-CEN protrusions on the cell periphery. Scanning confocal imaging of Ras activation (RBD, green) and actin polymerization (LimE_Δ *Coil*_, magenta) in AX3 *Dictyostelium* cells. Playback is 15 frames per second.
