# Supplementary material for: Using Live-Cell Imaging and Synthetic Biology to Probe Directed Migration in Dictyostelium
Source: Front Cell Dev Biol. 2021 Oct 5;9:740205. doi: 10.3389/fcell.2021.740205 (PMC8523838; doi:10.3389/fcell.2021.740205)
Supplement: Supplementary file 5 [file Data_Sheet_1.docx]

**Supplementary Methods**

Chemically induced dimerization

**Anchor design**

To target proteins to the membrane in *Dictyostelium*, a myristoylation sequence based on the first 150 amino acids of PKBR, a membrane-localized kinase, can be used (Miao et al., 2017). To create an anchor, attach this “myr” sequence to a tandem series of FKBP domains (2x FKBP) under the control of an *act15* promoter and express it in *Dictyostelium* using standard electroporation protocols (Chisholm et al., 2006). This myr-2X-FKBP system localizes uniformly to the plasma membrane of vegetative cells and is able to recruit FRB actuators in seconds to minutes (Miao et al., 2017). However, the myr sequence can show some preference for the back region of highly polarized cells (unpublished observations). Placing a tandem FKBP domain instead at the C-Terminus of cAR1, a membrane receptor which remains completely uniform across the membrane (Xiao et al., 1997), avoids this issue.

**Expression guidelines – vectors and fluorescent proteins**

For expressing CID systems and components, use a standard electroporation protocol to transform *Dictyostelium* and then inoculate them with heat-killed *Klebsiella Aerogenes* to increase survival. When choosing which vectors and fluorescent proteins to use in conjunction with an anchor or actuator, there are several important considerations. First, the expression level of the actuator must be high in order to create an observable change in cell behavior. To accomplish this, use the extrachromosomal vector pCV5 (Janetopoulos et al., 2001) or the integrating vector pB18 (Johnson et al., 1991), both of which contain a G418 selection marker. When imaging the actuator and anchor with no additional biosensors, a pB18 actuator is recommended due to its stable and high expression along with an anchor on an extrachromosomal vector carrying a hygromycin selectable marker like pDM358 (Veltman et al., 2009). Using a CID system with a biosensor requires a modified setup: express both the anchor and actuator on separate extrachromosomal G418 vectors (pCV5) and a biosensor on a hygromycin vector (pDM358). Transforming two extrachromosomal vectors with the same selectable marker at the same time provides sufficient expression of both the anchor and actuator (Miao et al., 2017). However, fewer cells with high expression of both components are observed, and more rapid loss of expression over time. In the future, dual-expression vectors may alleviate this issue (Veltman et al., 2009; Kim et al., 2011).

Fluorescent protein color choice is also important when designing CID constructs. While many imaging platforms support imaging in four colors with minimal bleedthrough, many live imaging experiments are limited to red and green. This is because far-red fluorescent proteins lack the brightness and stability of GFP, mCherry, and their derivatives (Lambert, 2019), and because the wavelength required to excite blue fluorescent proteins is extremely damaging to cells (Waldchen et al., 2015). As a convention, use the red channel (mCherry) to image actuators and confirm that they localize to the membrane after rapamycin addition. If there are no biosensors involved, attach an EGFP to the anchor to confirm anchor expression. However, it is often important to use the green channel to image biosensors. In these situations, either confirm anchor expression using blue or far-red proteins and limiting exposure to single frame or leave the anchor unlabeled and infer expression by actuator membrane localization. To image two separate biosensors, it would be necessary to move the actuator to the far-red channel or employ advanced imaging techniques like spectral deconvolution (Valm et al., 2017). Applying recent advances in cloning and expression technology like the GoldenBraid system to *Dictyostelium* may facilitate these three- and four-color experiments (Kundert et al., 2020).

**Experimental practice and controls**

There are a few general guidelines required for a successful CID experiment: 1) Image the cells for long enough prior to rapamycin addition to be able to quantify their baseline behavior, at least 10 minutes before membrane recruitment. 2) Add rapamycin quickly and evenly to ensure rapid recruitment. When using 8 well plates (LabTek II chambered coverglass, 0.7 cm^2^/well) with 450 μl of DB in each well add 50 μl of 50 μM Rapamycin in DB for a final concentration of 5 μM. Adding a large volume of media allows one to skip mixing after addition, which can move the imaging chamber or detach cells from the bottom surface. 3) Image long enough to observe both actuator membrane localization and phenotypic changes. Membrane localization typically occurs within two minutes, depending on expression and how well-distributed the rapamycin is in solution. The time it takes to observe a phenotype is highly dependent on the biology of the actuator and can be as short as a minute or as long as 30 minutes. When working with a new actuator, it is important to image for a long time to determine if and when a change occurs. 4) In order to obtain enough cells (30-50) for quantitative analysis acquire multiple stage positions if time allows and plate multiple wells to do several experiments.

Optogenetic control of cell activity

**Anchor and actuator design**

The same design principles described for the CID system apply when designing optogenetic systems. The highest expression and recruitment has been observed when attaching Cry2 or SSBP to the actuator, and CIBN or iLID to the anchor. To prevent dark-state recruitment in Dictyostelium, use SSBP_R73Q_, a mutant SSBP domain with a lower affinity for iLID in both the light state and dark state (Guntas et al., 2015). Finally, to confirm expression, it is important to visualize the actuator localizing to the membrane upon blue light exposure. This can be done by attaching the actuator to a red fluorescent protein. When imaging both the actuator and the biosensor (and anchor), attach one of them to a far-red protein

**Experimental practice and controls**

Many of the same guidelines and controls apply to optogenetics experiments as CID ones: Allow enough time before and after stimulation and confirm that there no off-target effects from the actuator. For optical stimulation, it is important to re-expose cells to blue light every 15-20 seconds for iLID, 3-4 minutes for Cry2 to maintain a consistent phenotype. Additionally, it is possible to test whether a phenotype is reversible by stopping blue right exposure. When doing local excitation on the membrane, shining light in a small (3-5 μm) region 1-2 μm outside of the cell produces the most precise localization. This may depend on the specifications of the targeted illumination system. For whole-cell experiments, it is simplest to acquire a full-field image using a 488 or 450 nm laser.

Experimental Guidelines for Analyzing Cells Quantitatively

Plate cells at 2 * 10^5^ cells/cm^2^ and lower the density afterwards if cells are too close together or increase it if there are too few cells expressing the desired markers. During acquisition, it is advisable to perform all experiments with a 1.45-1.49 NA oil immersion lens in order to accurately measure changes in cell shape. To maximize the signal-to-noise, use the lowest magnification or scan spacing that produces a pixel size of 110 nm (one half of the diffraction limit of visible light). Light intensity and exposure time should be as high as possible to image the cell for the time required without photobleaching or photodamage. To make vegetative Dictyostelium more resistant to photodamage, starve them by suspending them in DB buffer at 2 * 10^7^ cells/ml for 1-2 hours prior to imaging (Miao et al., 2017).

Analyzing Cell Behavior and Morphology

**Manual quantification**

To measure the speed of cells, identify their approximate centroid in each frame using a program like the Manual Tracking plugin, and then use any analysis software to calculate the displacement between frames. Depending on the goal of the experiment, the displacement can be used to calculate instantaneous velocity, average velocity, or more complex measurements like diffusion coefficients. Shape and area are also simple: use the Freehand Drawing Tool or the Polygon Selection Tool to identify the outline of the cell, and then use standard measuring functions to get important shape descriptors like area, perimeter, and roundness. For longer movies, save the outlined shapes to the ROI Manager to avoid losing work.

**Automatic quantification**

There are many approaches to cell segmentation, this section will only describe one basic outline: First, cropping the image to only include the analyzed cell makes segmentation much simpler. This can be done automatically using coordinates from a manual tracking program. Then, apply an intensity threshold to create a binary mask which only contains pixels where the label is bright. There are many methods for setting a threshold, but their effectiveness varies between samples; Otsu’s method (Otsu, 1979) or removing all pixels less than 1 standard deviation above the mean brightness are good starting points. A morphological closing operation can then remove small background dots outside the cell and close cracks in the cell mask. Finally, perform a filling operation to eliminate holes in the cell mask, and apply a size threshold to delete small bright objects. At this point, any image analysis platform can identify a single binary mask region as an object and extract geometric information like area. When applying this pipeline to a time series or multiple cells, it is important to check the shape of the mask as the program runs. A robust segmentation pipeline facilitates rapid quantification of many timepoints and cells. Additionally, analysis programs can extract more detailed information from the segmented data, like changes in shape, aspect ratio, and circularity.

**Supplementary Movie Legends**

**Movie S1: Increasing RacB and Rac1A activity using a CID system**

Scanning confocal imaging of an AX3 *Dictyostelium* cell (transmitted light, green) expressing a CID system designed to increase RacB and Rac1A activity. The cell is expressing cAR1-FKBP-FKBP (unlabeled) and mCherry-FRB-RacGEF1_ΔN_ (magenta), an activator of Rac1 fused to an FRB domain. After rapamycin addition, RacGEF1_ΔN_ is recruited to the membrane. Consistent with previous reports, the cell became very round with small, short-lived protrusions. t = 00:00 indicates rapamycin addition. Playback is 18 frames per second.

**Movie S2: The Cry2_PHR_-CIBN system in *Dictyostelium***

Scanning confocal imaging of Cry2_PHR_ membrane recruitment in AX3 *Dictyostelium* cells. Cells are also expressing an unlabeled membrane anchor, cAR1-CIBN. Yellow square indicates region illuminated with 488 nm light. t = 00:00 indicates blue light stimulation. Playback is 22 frames per second.

**Movie S3: Traveling STEN waves on the bottom of a giant cell**

Scanning confocal imaging of PIP3 (PH_Crac_, green) and a back protein (PTEN, magenta) in a giant AX2 *Dictyostelium* cell. Playback is 2 frames per second.

**Movie S4: STEN-CEN protrusions on the cell periphery**

Scanning confocal imaging of Ras activation (RBD, green) and actin polymerization (LimE_ΔCoil_, magenta) in AX3 *Dictyostelium* cells. Playback is 15 frames per second.

**References**

Chisholm, R. L., Gaudet, P., Just, E. M., Pilcher, K. E., Fey, P., Merchant, S. N., et al. (2006). dictyBase, the model organism database for Dictyostelium discoideum. *Nucleic Acids Res.* 34. doi:10.1093/nar/gkj090.

Guntas, G., Hallett, R. A., Zimmerman, S. P., Williams, T., Yumerefendi, H., Bear, J. E., et al. (2015). Engineering an improved light-induced dimer (iLID) for controlling the localization and activity of signaling proteins. *Proc. Natl. Acad. Sci. U. S. A.* 112, 112–117. doi:10.1073/pnas.1417910112.

Janetopoulos, C., Jin, T., and Devreotes, P. (2001). Receptor-mediated activation of heterotrimeric G-proteins in living cells. *Science (80-. ).* 291, 2408–2411. doi:10.1126/science.1055835.

Johnson, R. L., Caterina, M. J., Devreotes, P. N., Van Haastert, P. J. M., and Vaughan, R. A. (1991). Overexpression of the cAMP Receptor 1 in Growing Dictyostelium Cells. *Biochemistry* 30, 6982–6986. doi:10.1021/bi00242a025.

Kim, J. H., Lee, S. R., Li, L. H., Park, H. J., Park, J. H., Lee, K. Y., et al. (2011). High cleavage efficiency of a 2A peptide derived from porcine teschovirus-1 in human cell lines, zebrafish and mice. *PLoS One* 6. doi:10.1371/journal.pone.0018556.

Kundert, P., Sarrion-perdigones, A., Gonzalez, Y., Katoh-kurasawa, M., Hirose, S., Lehmann, P., et al. (2020). A GoldenBraid cloning system for synthetic biology in social amoebae. 48, 4139–4146. doi:10.1093/nar/gkaa185.

Lambert, T. J. (2019). FPbase: a community-editable fluorescent protein database. *Nat. Methods* 16, 277–278. doi:10.1038/s41592-019-0352-8.

Miao, Y., Bhattacharya, S., Edwards, M., Cai, H., Inoue, T., Iglesias, P. A., et al. (2017). Altering the threshold of an excitable signal transduction network changes cell migratory modes. *Nat. Cell Biol.* 19, 329–340. doi:10.1038/ncb3495.

Otsu, N. (1979). Threshold selection method from gray-level histograms. *IEEE*

*Trans. Syst. Man Cybern*. 9, 62–66. doi: 10.1109/tsmc.1979.4310076

Valm, A. M., Cohen, S., Legant, W. R., Melunis, J., Hershberg, U., Wait, E., et al. (2017). Applying systems-level spectral imaging and analysis to reveal the organelle interactome. *Nature* 546, 162–167. doi:10.1038/nature22369.

Veltman, D. M., Akar, G., Bosgraaf, L., and Van Haastert, P. J. M. (2009). A new set of small, extrachromosomal expression vectors for Dictyostelium discoideum. *Plasmid* 61, 110–118. doi:10.1016/j.plasmid.2008.11.003.

Waldchen, S., Lehmann, J., Klein, T., Van De Linde, S., and Sauer, M. (2015). Light-induced cell damage in live-cell super-resolution microscopy. *Sci. Rep.* 5. doi:10.1038/srep15348.

Xiao, Z., Zhang, N., Murphy, D. B., and Devreotes, P. N. (1997). Dynamic distribution of chemoattractant receptors in living cells during chemotaxis and persistent stimulation. *J. Cell Biol.* 139, 365–374. doi:10.1083/jcb.139.2.365.
